# The Effect of Lung Resection for NSCLC on Circulating Immune Cells: A Pilot Study

**DOI:** 10.3390/curroncol30050387

**Published:** 2023-05-17

**Authors:** Joseph D. Phillips, Kayla A. Fay, Alan J. Bergeron, Peisheng Zhang, Daniel W. Mielcarz, Andrew M. Calkins, Tyler G. Searles, Brock C. Christensen, David J. Finley, Mary Jo Turk, Jacqueline Y. Channon

**Affiliations:** 1 Department of Surgery, Dartmouth-Hitchcock Medical Center, The Geisel School of Medicine at Dartmouth, Lebanon, NH 03756, USA; 2 DartLab, Dartmouth Cancer Center, Lebanon, NH 03756, USA; 3 Department of Microbiology and Immunology, The Geisel School of Medicine at Dartmouth, Lebanon, NH 03756, USA; 4 Departments of Epidemiology and Molecular & Systems Biology, The Geisel School of Medicine at Dartmouth, Lebanon, NH 03756, USA

**Keywords:** non-small cell lung cancer, lung resection, regulatory T cells, circulating immune cells, Treg subsets, single-cell sequencing

## Abstract

This pilot study sought to evaluate the circulating levels of immune cells, particularly regulatory T-cell (Treg) subsets, before and after lung resection for non-small cell lung cancer. Twenty-five patients consented and had specimens collected. Initially, peripheral blood of 21 patients was collected for circulating immune cell studies. Two of these patients were excluded due to technical issues, leaving 19 patients for the analyses of circulating immune cells. Standard gating and high-dimensional unsupervised clustering flow cytometry analyses were performed. The blood, tumors and lymph nodes were analyzed via single-cell RNA and TCR sequencing for Treg analyses in a total of five patients (including four additional patients from the initial 21 patients). Standard gating flow cytometry revealed a transient increase in neutrophils immediately following surgery, with a variable neutrophil–lymphocyte ratio and a stable CD4–CD8 ratio. Unexpectedly, the total Treg and Treg subsets did not change with surgery with standard gating in short- or long-term follow-up. Similarly, unsupervised clustering of Tregs revealed a dominant cluster that was stable perioperatively and long-term. Two small FoxP3^hi^ clusters slightly increased following surgery. In the longer-term follow-up, these small FoxP3^hi^ Treg clusters were not identified, indicating that they were likely a response to surgery. Single-cell sequencing demonstrated six CD4+FoxP3+ clusters among the blood, tumors and lymph nodes. These clusters had a variable expression of FoxP3, and several were mainly, or only, present in tumor and lymph node tissue. As such, serial monitoring of circulating Tregs may be informative, but not completely reflective of the Tregs present in the tumor microenvironment.

## 1. Introduction

Lung cancer remains the leading cause of cancer-related mortality worldwide [1]. Improving the immune system’s ability to fight cancer continues to be an area of intense research, particularly with the success of immunotherapies in melanoma and non-small cell lung cancer (NSCLC) [2,3]. The characterization of host immune cell parameters prior to treatment may identify the biomarkers predictive of clinical outcomes and response to immunotherapy [4]. For example, a pretreatment circulating neutrophil-to-lymphocyte ratio (NLR) ≥ 2.5 has been associated with worse survival in NSCLC [5,6]. Regulatory T cells (Treg) are CD4+CD25+Forkhead box P3 (FoxP3)+ lymphocytes that maintain self-tolerance and immune homeostasis, and are known to infiltrate the tumor microenvironment (TME) and suppress cancer-associated inflammation [7]. An increased Treg infiltration in tumors is associated with recurrence and worse survival in NSCLC [4,8]. Moreover, circulating Treg in the peripheral blood have been shown to be increased in NSCLC patients compared to healthy controls [9,10,11,12,13].

Tregs can be induced centrally in the thymus or generated in the periphery through the conversion of naïve CD4+ T cells to Tregs [14,15,16]. Peripheral Tregs are thought to be the main type of Tregs in the TME of most cancers [17]. Circulating Tregs in the blood may be reflective of the TME. Blood Tregs with an increased expression of immune checkpoint molecules, such as PD-1, have been found in cancer patients in contrast to healthy controls [18]. However, the highest density of Tregs with increased immune checkpoint molecules is found in the TME, indicating that these cells exist in heterogeneous populations across tissue compartments. The engagement of the PD-1–PD-L1 axis is one of the mechanisms through which Tregs suppress effector T cells, and treatments that reduce the suppression of cancer-associated inflammation by inhibiting Tregs would potentially limit tumorigenesis and/or enhance immunotherapy responses [14,19].

While most studies of peripheral blood evaluate the circulating levels of total Tregs, subpopulations with functional and phenotypic delineations have been demonstrated in inflammatory diseases, colon cancer and lung cancer [10,11,20,21,22]. There are numerous ways to categorize the heterogeneity of Treg subsets by their developmental stage and phenotypic markers [20,23], which can make comparisons between studies and nomenclature challenging. Nevertheless, from a clinical perspective, higher levels of circulating Treg subsets have been associated with worse overall survival in lung cancer patients and may represent a potential prognostic factor or biomarker for treatment response [11,24]. 

While there is some evidence of the clinical importance of Treg subsets in NSCLC, the immediate and longer-term effects of surgery on their circulating levels are less studied. This pilot study of 25 patients sought to evaluate the changes in circulating immune cells, particularly Treg subsets, during the perioperative period (n = 21). As the TME is thought to be the stimulus for increased circulating Tregs in cancer, we hypothesized that Tregs would be elevated prior to surgery and decreased in the post-operative period. We evaluated changes in circulating immune cells during the perioperative period and focused on Tregs at longer time points in a subset of these patients with available data (n = 10), using standard gating and unsupervised clustering flow cytometry analyses. To investigate the composition of Tregs across tissue compartments, we performed single-cell RNA sequencing (scRNA-seq) and single-cell T-cell receptor sequencing (scTCR-seq) of the tumors, lymph nodes and peripheral blood of five patients.

## 2. Materials and Methods

### 2.1. Patients

Patients with known or suspected NSCLC undergoing surgical resection for a planned standard of care treatment were eligible for study inclusion. Exclusion criteria included history of another malignancy, prior systemic therapy or radiation, history of autoimmune disease, or actively receiving immunosuppressive drugs, such as steroids. Lung malignancies were staged according to the AJCC TNM classification 8th edition. All patients signed an informed consent form for the study. The study was approved by the Committee for the Protection of Human Subjects of Dartmouth–Hitchcock Medical Center. 

### 2.2. Peripheral Blood Samples and Flow Cytometry

The peripheral blood samples from patients were collected in heparinized vacutainers (n = 25), with 21 patients consenting for the circulating immune cell studies (Supplemental Figure S1). Due to technical issues (n = 2), 19 patients had analyzable samples immediately prior to surgery (pre-resection) and ~1 h after surgery in the post-anesthesia care unit (post-resection). Eighteen patients had matched samples from their pre-resection, post-resection and 2-week post-operative follow-up visit. Samples were also collected at 6, 12 and 18 months after surgery, though only in a subset of 10 patients owing to disruptions in clinical surveillance related to the COVID-19 pandemic. Four additional patients had blood samples matched with the tumor and lymph node tissues collected for single-cell scRNA-seq and scTCR-seq, as described below. Sample processing occurred as soon as possible after collection, typically within 1–2 h. Peripheral blood samples utilized whole blood and were stained with two separate panels: 1) leukocyte panel: CD45, CD3, CD4, CD8, CD19, CD56, CD14 and CD16; and 2) Treg panel: CD45, CD3, CD4, CD45RA, CD25, CD146, FoxP3, RORγt, CTLA-4 (CD152), PD-1 (CD279), IL-17 and IL-10. (Supplemental Table S1). The panels were pre-optimized for antibody titer and staining conditions, and fluorescence-minus-one (FMO) controls were used to assess the fluorescence spread. Flow cytometry analyses were carried out in DartLab, the Immune Monitoring and Flow Cytometry Shared Resource at the Dartmouth Cancer Center. Aliquots of whole blood were stained immediately after blood draw with the leukocyte panel for 30 min at room temperature, followed by red blood cell lysis with the BD FACS Lysing solution (BD Biosciences), and washing. Flow cytometry was performed on a 5-laser, 27-color ZE5 flow cytometer (Bio-Rad). The CD-Chex cells (Streck) were stained in parallel to control for inter-day variability. Standard flow cytometry manual gating strategies for the leukocyte panel were performed using the FlowJo v10.8.2 software (BD Biosciences) (Supplemental Figure S2). 

Additional aliquots of whole blood were unstimulated or stimulated for 4 h with PMA (100 ng/mL) and Ionomycin (2 μg/mL) with monensin (3 μM) added after 1 h, and stained with the cell surface Treg panel markers in the presence of the Brilliant Stain Buffer (BD Biosciences). After red blood cell lysis and overnight permeabilization with the FoxP3 Fix/Perm buffer (BioLegend) at 4 °C, the cells were stained with intracellular Treg panel markers for 45 min at room temperature, then washed. Cryopreserved peripheral blood mononuclear cells from a healthy donor were stained in parallel to control for inter-day variability. 

Standard flow cytometry manual gating strategies for CD4+ Treg subgroups were performed, as reported by Miyara et al. [20]: Fr I (resting Treg): CD45RA+FoxP3+CD25+, Fr II (activated Treg): CD45RA-FoxP3++CD25+, Fr III (mixture of Treg and non-Treg): CD45RA-FoxP3+CD25+ (Supplemental Figure S3). Unsupervised clustering was carried out using the software package R, time-of-flight mass cytometry (CyTOF) workflow [25], Flow Self-Organizing Map (FlowSOM) [26], ConsensusClusterPlus [27,28] and UMAP packages [29]. Single-cell CD45+ cell populations from the manual analysis were used to determine CD3+ clusters, that were then re-clustered to evaluate CD4+ cell populations to identify FoxP3+ clusters in an unbiased manner, as previously described [25]. Initial clustering was performed using patient samples with pre-resection, post-surgical and 2-week time point data (57 samples from 19 individual patients). Long-term clustering was performed using patient samples at the 6-, 12- and 18-month time points (18 samples from 10 individual patients). 

### 2.3. Tissue Processing for scRNA-Seq and scTCR-Seq

All tissues for scRNA-seq and scTCR-seq were collected and processed the same day (n = 5). The tumor and lung samples were minced into small pieces using surgical scissors, and suspended in 5 mL of HBSS with Ca^2+^ and Mg^2+^ containing 1450 units/mL of collagenase type IV (Worthington Biochemical, Lakewood, NJ, USA) and 0.2 mg/mL DNase I (Sigma, St. Louis, MO, USA) in a 15 mL vial. These tissues were then incubated at 37 °C on a rotating rack for 30 min. The lymph nodes were processed similarly, with a collagenase concentration diluted to 725 mg/mL in 3 mL and an incubation time of 15 min. After incubation, the samples were diluted in a cold flow buffer (PBS containing 2% heat inactivated FBS) and strained through a 70 μm cell strainer. The samples were then centrifuged at 1800 rpm for 8 min and resuspended in a cold flow buffer for staining. PBMCs were isolated from whole blood using the Ficoll-Paque™ PLUS Media (Cytiva, Marlborough, MA, USA) density gradient.

### 2.4. Staining and FACS Sorting for Single-Cell Analysis

Single-cell suspensions were Fc-blocked using Human TruStain FcX (Biolegend, 1:20 dilution) for 15 min on ice. The cells were then stained at 1:100 with anti-CD45 BV711 (Biolegend, San Diego, CA, USA), anti-CD8 APC (Biolegend) and anti-CD4 FITC (Biolegend) antibodies for 30 min in the dark on ice. Each individual tissue was stained with a unique hashtag antibody (Biolegend) to allow for the samples to be multiplexed on a scRNA-seq lane. After staining, the cells were washed with 2 mL of cold flow buffer twice to remove any unbound antibodies and resuspended in flow buffer containing DAPI (Biolegend), to stain the dead cells. FACS was performed on a Sony SH800 cell sorter. The cells were sorted until 5000 CD4 cells were acquired from each tissue and blood sample.

### 2.5. scRNA-Seq and scTCR-Seq Profiling

Following sorting, the cells were submitted for scRNA and scTCR sequencing following the 10× Genomics guidelines, as previously described [30]. The cells were submitted via 10× Chromium Chip G lane. Following the RNA and TCR V(D)J library construction using the Chromium Single Cell 5′ Library and Gel Bead Kit (10× Genomics), and Illumina sequencing, raw sequencing data were run through the Cell Ranger v6 pipeline (10× genomics) using the human reference genome GRCh38, to produce the gene expression matrices for scRNA-seq and CSVs-containing TRA/TRB T cell receptor sequences for each cell.

### 2.6. scRNA-Seq and scRCR-Seq Data Processing

The gene expression matrices from the Cell Ranger output were processed using the R package Seurat (v4). Low-quality cells were excluded by removing cells with less than 500 genes or more than 12.5 percent mitochondrial genes. To accumulate T cells, the cells expressing CD79A or CD68 were removed. Data from each 10× Genomics sample were individually normalized and scaled using the ‘SCTransform’ function. All samples were then integrated together following the Satija Lab’s Seurat integration vignette. To focus on Tregs, the cells not expressing *FOXP3* were removed at this point. For both integration and the following principal component (PC) analysis, TCR genes were removed from the variable gene list to prevent clustering based on these transcripts. The remaining cells were clustered using the Seurat functions ‘FindNeighbors’ and ‘FindClusters’, using the first 12 PCs. These 12 PCs were also used to generate the UMAP plots of the clusters.

### 2.7. Statistical Analysis

Statistical analysis was performed using the Prism software package (version 9.4.0, GraphPad Software Inc, San Diego, CA, USA). The differences between time points were assessed using paired Student’s *t*-tests. Two-sided *p*-values < 0.05 were considered statistically significant.

## 3. Results

### 3.1. Patient Characteristics

Twenty-five patients with diagnosed or suspected NSCLC consented for the study and had their samples collected (Supplemental Figure S1); all were treatment-naive. For the circulating immune cell studies, 21 patients had their blood drawn. Due to technical issues, 19 patients had at least one pre-resection and post-resection sample and were analyzed. Eighteen patients had a matched pre-resection, post-resection and 2-week sample for the circulating leukocyte analysis. Of these 18 patients, 1 also had a matched tumor and lymph node for scRNA-seq and scTCR-seq studies. Four additional patients were included for the scRNA-seq and scTCR-seq studies only. The baseline clinical characteristics of the 25 consenting patients are presented in Table 1. Twenty-four patients with malignancy (including one carcinosarcoma) and one patient with a benign granuloma were consented. As this was a pilot study with somewhat small numbers, we included all patients with analyzable data. 

### 3.2. Circulating Leukocytes via Standard Flow Cytometry Gating

**Figure 1:**
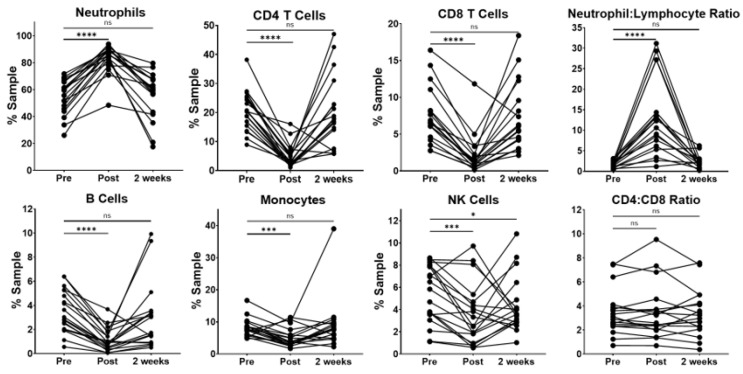
Lung resection results in the transient increase in neutrophils, but a stable CD4–CD8 ratio. Matched dot plots of each cell type, the neutrophil-to-lymphocyte ratio (NLR) and CD4-to-CD8 ratio at three time points, from prior to surgery (Pre, n = 19), immediately following surgery (Post, n = 19) and 2 weeks after surgery (2 weeks, n = 18). Dots connected by lines indicate individual patients. There is a statistically significant increase in the percentage of neutrophils immediately following surgery and a corresponding decrease in all other cells types. These frequencies return to baseline for all cell types 2 weeks after surgery, except for a slight continued decrease in NK cells (mean 5.4% vs. 4.4%, *p* < 0.05). There is a transient, variable increase in the NLR in patients immediately after surgery, with no significant change by 2 weeks. The CD4–CD8 T cell ratio remained relatively stable in patients across the three time points. Significance determined via paired Student’s *t* tests. ns: non-significant, * *p* < 0.05, *** *p* < 0.001, **** *p* < 0.0001.

For the circulating immune cells, patients exhibited a wide variability in the percentage of neutrophils at the pre-resection time point. (Figure 1) As expected, the surgical resection was related to an immediate and transient increase in the percentage of peripheral blood neutrophils. Interestingly, neutrophils returned to pre-resection levels 2 weeks after surgery. The observed increase in neutrophils was related with significant decreases in the percentages of CD4 and CD8 T cells, B cells, monocytes and NK cells at the immediate post-resection time point. However, when compared to pre-resection, these populations showed no statistically significant difference at the 2-week time point, with the exception of NK cells, which remained slightly decreased in a majority of the patients. 

Because the pretreatment neutrophil–lymphocyte ratio (NLR) has been associated with survival in NSCLC [5,6], we evaluated this in our patient cohort. We observed a large, variable increase in the NLR at the immediate post-resection time point compared to pre-resection. The NLR was not significantly different compared to baseline levels by the 2-week time point. Interestingly, the CD4-to-CD8 ratio remained relatively stable across the three perioperative time points, indicating that the total CD4 T cells did not significantly change relative to the other lymphocytes in our patient cohort. 

### 3.3. Circulating Tregs via Standard Flow Cytometry Gating

**Figure 2:**
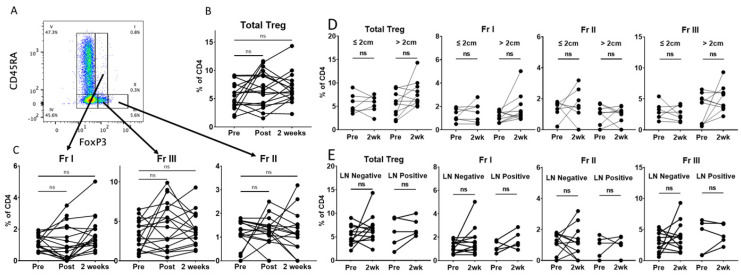
Circulating Treg proportions are stable within 2 weeks of lung tumor resection. (**A**). Representative manual gating strategy for peripheral blood CD45+CD3+CD4+ cells with the Treg panel to identify the Treg subsets, as described by Miyara et al., 2009 [20]. (**B**). Matched dot plots demonstrating no significant difference immediately post-surgery (n = 18) or at 2 weeks (n = 17) for total Tregs. (**C**). Matched dot plots demonstrating no significant difference in the Treg subsets post-operatively or 2 weeks after surgery. (**D**). Matched dot plots demonstrating no significant difference in total Tregs or the Treg subsets 2 weeks after surgery when stratified by tumor ≤2 cm (n = 7) vs. >2 cm (n = 10). (**E**). Matched dot plots demonstrating no significant difference in total Tregs or the Treg subgroups 2 weeks after surgery when stratified by LN-negative (n = 12) and LN-positive (n = 5) tumors. Dots connected by lines indicate individual patients. Fr I (resting Treg): CD45RA+FoxP3+CD25+, Fr II (activated Treg): CD45RA-FoxP3++CD25+, Fr III (mixture of Treg and non-Treg): CD45RA-FoxP3+CD25+. Significance determined via paired Student’s *t* tests. ns: non-significant.

Having demonstrated stable levels of total CD4 T cells between the preoperative and 2-week time points, we sought to investigate the changes in circulating levels of CD4 Tregs and Treg subsets, as described by Miyara et al. [20]. These Treg fractions are subdivided by their expression of CD45RA and FoxP3: Fr I (resting Treg) CD45RA+FoxP3+CD25+, Fr II (activated Treg) CD45RA-FoxP3++CD25+ and Fr III (mixture of Treg and non-Treg) CD45RA-FoxP3+CD25+ (Figure 2A and Supplemental Figure S3). Contrary to our hypothesis, we found no statistically significant difference in the proportion of total Tregs at the post-resection or 2-week time points using standard flow cytometry gating (Figure 2B). Furthermore, we saw no significant change in the proportions of Fr I, Fr II or Fr III Tregs at these perioperative time points (Figure 2C). Interestingly, there appeared to be a variable response among individual patients. We hypothesized that this could relate to the tumor size or lymph node involvement. However, we found no difference in total Tregs or FrI, FrII or FrIII Treg subsets when patients were stratified by tumor size (Figure 2D) or lymph node involvement (Figure 2E). Circulating Tregs have been reported to be increased in smokers and patients with COPD [31]. In our cohort, we did not observe a difference in total Tregs or the Treg subsets prior to resection when patients were stratified by smoking status (former vs. current) or COPD (Supplemental Figure S4). No significant change was observed for smoking status at the 2-week time point. Interestingly, patients without COPD had a small increase in total Tregs at the 2-week time point, which was not driven by a statistically significant change in Fr I, Fr II or Fr III. This seemed to be increased in a single patient, who was a current smoker without COPD, and a skewed result is possible, given the small size of our cohort.

**Figure 3:**
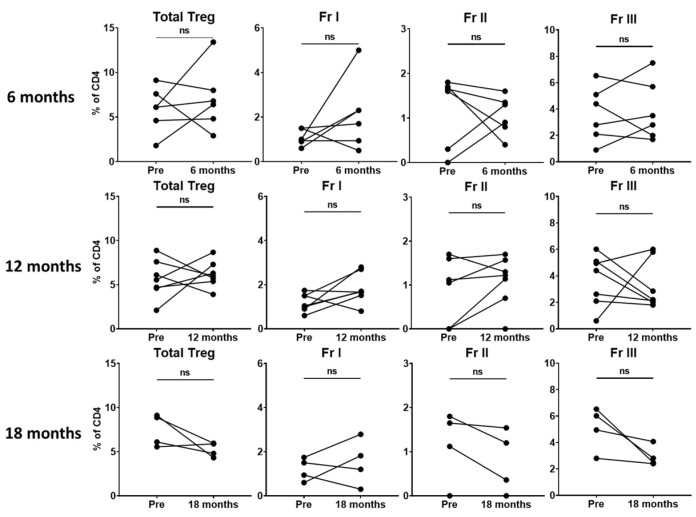
Circulating Treg proportions do not significantly change long-term following surgery. Matched dot plots demonstrating no significant differences in total Tregs or the three Treg fractions at 6 months (n = 6), 12 months (n = 7) or 18 months (n = 4) in patients with available data. Dots connected by lines indicate individual patients. Fr I (resting Treg): CD45RA+FoxP3+CD25+, Fr II (activated Treg): CD45RA-FoxP3++CD25+, Fr III (mixture of Treg and non-Treg): CD45RA-FoxP3+CD25+. Significance determined via paired Student’s *t* tests. ns: non-significant. Data are from 10 individual patients.

Given that we did not find significant changes in total Tregs or Treg subsets within the perioperative period, we hypothesized that 2 weeks may not be sufficient time to observe a meaningful change. Thus, we evaluated patients with available later follow-up data to assess potential differences in circulating Treg levels at longer-term time points. Unfortunately, due to the COVID-19 pandemic, routine surveillance visits and research specimen collection were sporadic. In the small number of patients with available data (n = 10), we found no significant difference in total Tregs or the Treg subsets in patients at 6 (n = 6), 12 (n = 7) or 18 months (n = 4) (Figure 3). Two patients in our cohort had a recurrence, both at 18 months. While data related to recurrence would be extremely informative, unfortunately, we were unable to capture a sample at this time point for these patients due to COVID-19 pandemic issues.

### 3.4. Circulating Tregs via Unsupervised Clustering

The standard flow cytometry gating of Treg fractions can be labor- and time-intensive and subjective, and these subsets may be made of further subpopulations of cells [32]. Recently, advances in high-dimensional flow cytometry analyses using computer algorithms allow more sophisticated unsupervised gating strategies that cluster cells in an unbiased, objective manner, based on the expression of multiple markers rather than a sequential expression of single markers [25,33,34]. To evaluate the changes in circulating Tregs in a potentially more objective manner, we applied this strategy to data from our patient cohort. 

**Figure 4:**
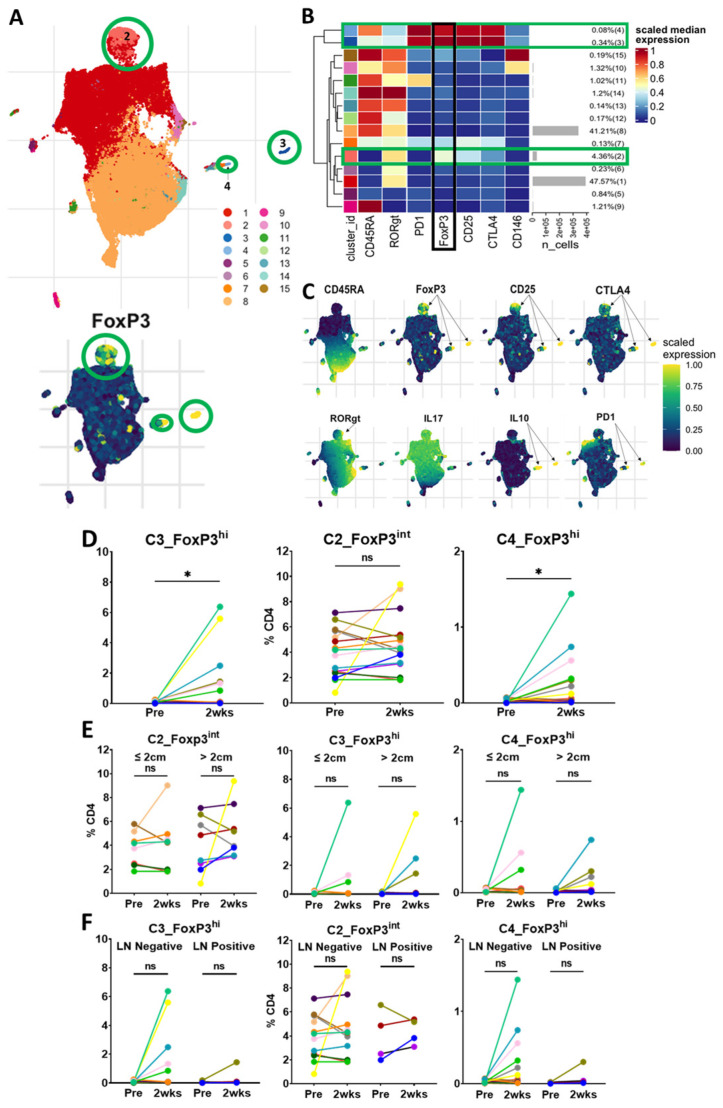
Unsupervised clustering of unstimulated cells reveals three CD4+ Foxp3-expressing clusters. (**A**). UMAPs of the unsupervised clustering of patients of concatenated files of unstimulated CD4+ cells at the pre-resection, post-resection and 2-week time points, n = 19 individual patients. In the upper UMAP, each cluster has a corresponding number and color. The lower UMAP demonstrates the relative expression of FoxP3. Green circles highlight the three Treg clusters (2, 3 and 4). (**B**). Heatmap demonstrating the relative scaled median marker expression, cell number and percentage of the total sample of each of the fifteen CD45+CD4+ clusters. Clusters 2, 3 and 4 have expression of FoxP3, CD25 and CTLA-4, indicating that they are Tregs. Green bars highlight the three Treg clusters. The black bar highlights the relative expression of FoxP3. (**C**). UMAPs demonstrating the scaled, relative expression of additional markers used to generate the fifteen CD4+ clusters. Black arrows highlight the Treg clusters with the expression of the separate markers used. (**D**). Matched dot plots (n = 16 patients) demonstrating no significant change in the predominant cluster, C2_FoxP3^int^, with a significant increase in the two small FoxP3hi clusters (C3_FoxP3^hi^ and C4_FoxP3^hi^), two weeks after surgery. (**E**). Matched dot plots demonstrating no significant change in the three unsupervised Treg clusters when stratified by tumor size at two weeks after surgery (tumor ≤ 2 cm, n = 8; tumor > 2 cm, n = 8). (**F**). Matched dot plots demonstrating no significant change in the three unsupervised Treg clusters when stratified by lymph node status at two weeks after surgery (LN negative, n = 12; LN positive, n = 4). Dots connected by lines indicate individual patients, each color is an individual patient. Significance determined via paired Student’s *t* tests. ns: non-significant, * *p* < 0.05. LN: lymph node.

Unsupervised clustering analysis of CD45+CD3+CD4+ cells revealed three FoxP3+ Treg clusters with one predominant cluster (C2_FoxP3^int^) and two smaller clusters (C3_FoxP3^hi^ and C4_FoxP3^hi^) (Figure 4A–C). C2_FoxP3^int^, which comprised 4.36% of the total CD4 population, was intermediate in the expression of FoxP3, CD45RA-negative and intermediate in the expression of CD25, CTLA-4 and RORγt, indicating that cells in this cluster are similar to Fr III from standard gating, and likely a mix of Tregs and non-Tregs [10,20]. On matched-patient analysis, cells in C2_FoxP3^int^ did not change significantly within 2 weeks of surgery or vary by tumor size or lymph node status. (Figure 4D–F).

C3_FoxP3^hi^, which comprised 0.34% of the CD4 population, was high in the expression of FoxP3, CD45RA-negative, high in the expression of CD25, CTLA-4 and PD-1, and low in the expression of CD146. The cells in C3_FoxP3^hi^ are similar to Fr II from the standard gating strategy and likely activated Tregs. Interestingly, the cells in C3_FoxP3^hi^ were present in very small proportions prior to resection and significantly increased 2 weeks following surgery. This change did not seem to be related to the tumor size or lymph node status. 

The cells in C4_FoxP3^hi^ were also present at a low frequency prior to surgery and significantly increased 2 weeks after resection. These cells were high in the expression of FoxP3, CD45RA-positive, with a high expression of CD25, CTLA-4 and PD-1, and a low expression of CD146. These cells may be in a transitional state from resting Treg to activated Treg. As a small CD45RA+Foxp3^hi^ population, they would potentially be missed in standard gating analysis. C4_FoxP3^hi^ did not seem to be affected by the tumor size or lymph node status in our cohort. Ultimately, unsupervised clustering revealed similar results to standard gating, demonstrating three FoxP3+ populations. The majority of these cells clustered in a FoxP3 intermediate population that did not decrease with surgery. Interestingly, we found two small clusters with a high expression of FoxP3, which, contrary to our hypothesis, increased following surgery. 

### 3.5. Circulating Tregs via Unsupervised Clustering with In Vitro Stimulation

**Figure 5:**
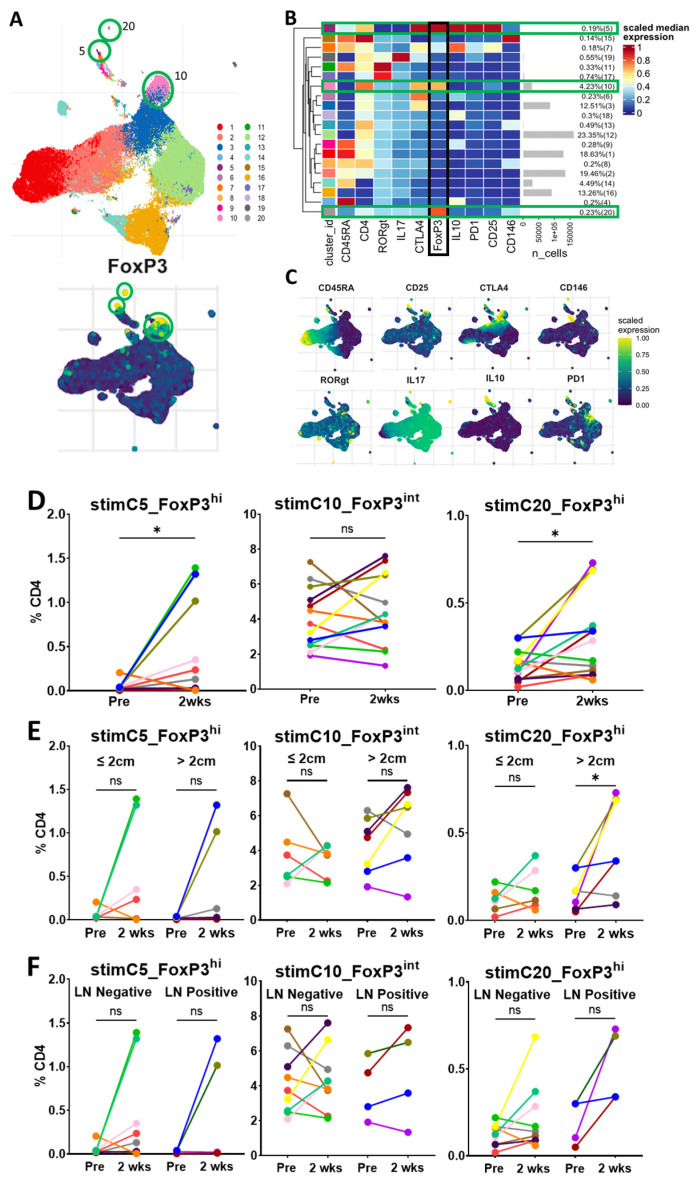
Unsupervised clustering of stimulated cells reveals three CD4+ Foxp3-expressing clusters. (**A**). UMAPs of unsupervised clustering of patients of concatenated files of CD4+-stimulated cells with PMA/Ionomycin at the pre-resection, post-resection and 2-week time points, n = 19 individual patients. In the upper UMAP, each cluster has a corresponding number and color. The lower UMAP demonstrates the relative expression of FoxP3. Green circles highlight the three Treg clusters (5, 10 and 20). (**B**). Heatmap demonstrating the relative scaled median marker expression, cell numbers and percentage of the total concatenated sample of each of the 20 CD4+ clusters of stimulated cells. Clusters 5, 10 and 20 have expression of FoxP3, CD25 and CTLA-4 indicating that they are Tregs. Green bars highlight the three Treg clusters. The black bar highlights the relative expression of FoxP3. (**C**). UMAPs demonstrating the scaled, relative expression of markers used to generate the 20 clusters. (**D**). Matched dot plots (n = 13) demonstrating a significant increase in clusters stimC5_FoxP3^hi^ and stimC20_FoxP3^hi^, with no significant change in stimC10_FoxP3^int^ at 2 weeks after surgery. (**E**). Matched dot plots demonstrating no significant change in clusters stimC5_FoxP3^hi^ and stimC10_FoxP3^int^, but an increase in larger tumors in stimC20_FoxP3^hi^ when stratified by tumor size at 2 weeks after surgery (tumor ≤ 2 cm, n = 6; tumor > 2 cm, n = 7). (**F**) Matched dot plots demonstrating no significant change in the three stimulated Treg clusters when stratified by lymph node status at 2 weeks after surgery (LN-negative, n = 9; LN-positive, n = 4). Dots connected by lines indicate individual patients, each color is an individual patient. Significance determined via paired Student’s *t* tests. ns: non-significant, * *p* < 0.05. LN: lymph node.

Having found phenotypically similar populations to standard gating with unsupervised clustering, we investigated the potential changes in the functional state of Tregs after surgery. We performed analyses and the unsupervised clustering of cells that had undergone stimulation in vitro with PMA and ionomycin, which activates cytokine production. Thirteen patients had matched pre-resection- and 2-week time-point stimulated data for analysis. As expected, the clustering of the stimulated cells also revealed three distinct FoxP3+ populations, with a predominant cluster presenting an intermediate expression of FoxP3 (stimC10_FoxP3^int^) and two small clusters with a high FoxP3 expression (stimC5_FoxP3^hi^ and stimC20_FoxP3^hi^) (Figure 5A–C). The cells in stimC10_FoxP3^int^ were CD45RA-negative, with a relatively low expression of CD25, RORγt and IL-17. These cells are likely the stimulated version of C2_FoxP3^int^ from the unstimulated clustering, and a mix of Treg and cytokine-producing non-Treg cells [10,20]. The cells in stimC10_FoxP3^int^ did not change significantly within 2 weeks of surgery or vary by tumor size or lymph node status (Figure 5D–F).

The cells in stimC5_FoxP3^hi^ were CD45RA-negative, with a high expression of CD25, CTLA-4, PD-1 and IL-10. These cells are likely the stimulated version of C3_FoxP3^hi^ from the unstimulated clustering, and produce IL-10, consistent with activated Tregs [20]. Consistent with the unstimulated clustering, the cells in stimC5_FoxP3^hi^ were significantly increased 2 weeks after surgery, particularly in a subset of patients, but did not vary with tumor size or lymph node status. 

The cells in stimC20_FoxP3^hi^ were CD45RA-negative, with a low expression of CD25, CTLA-4, IL-17, RORγt and CD146. These cells may be a small subset of cells transitioning between Fr III and Fr II in standard gating. In a notable subset of patients, the population of cells in stimC20_FoxP3^hi^ was also present at low levels prior to resection, and significantly increased 2 weeks following surgery. Interestingly, the 2-week increase in stimC20_FoxP3^hi^ was noted in patients with larger tumors, but was not different when stratified by lymph node status. 

Based on the unsupervised clustering of unstimulated and in-vitro stimulated cells, we hypothesized that the increases observed in the two small clusters of FoxP3^hi^ cells (C3_FoxP3^hi^ and C4_FoxP3^hi^ for unstimulated cells and stimC5_FoxP3^hi^ and stimC20_FoxP3^hi^ for stimulated cells) identified 2 weeks after the resection may be related to the general inflammatory response to surgery, rather than a reflection of the changes from tumor excision.

### 3.6. Circulating Tregs via Unsupervised Clustering of Long-Term Patients

**Figure 6:**
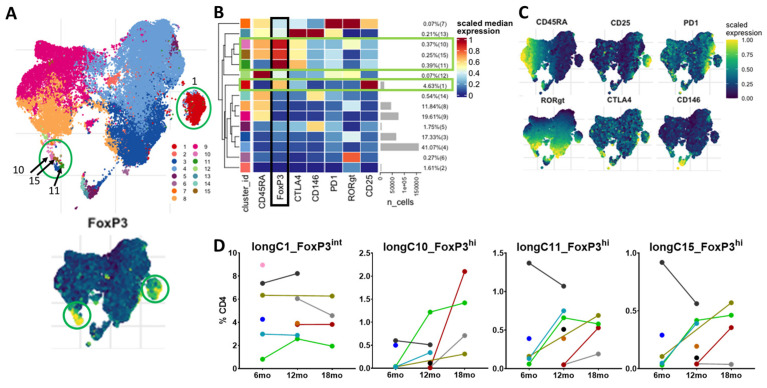
Unsupervised clustering of unstimulated cells from long-term time points reveals a predominant CD4+ FoxP3^int^-expressing cluster and three small FoxP3^hi^ clusters. (**A**). UMAPs of the unsupervised clustering of the concatenated files of CD45+CD3+CD4+ cells at the 6-, 12- and 18-month time points following surgery in 10 individual patients. In the upper UMAP, each cluster has a corresponding number and color. The lower UMAP demonstrates the relative expression of FoxP3. Green circles highlight the four Treg clusters (longC1_FoxP3^int^, longC10_FoxP3^hi^, longC11_FoxP3^hi^ and longC15_FoxP3^hi^). (**B**). Heatmap demonstrating the relative scaled median marker expression, cell numbers and percentages of the total sample of each of the fifteen CD45+CD3+CD4+ clusters. Clusters longC1_FoxP3^int^, longC10_FoxP3^hi^, longC11_FoxP3^hi^, and longC15_FoxP3^hi^ have the expression of FoxP3, CD25 and CTLA-4, indicating that they are Tregs. Green bars highlight the four Treg clusters. The black bar highlights the relative expression of FoxP3. (**C**). UMAPs demonstrating the scaled, relative expression of markers used to generate the 15 clusters. (**D**). Matched dot plots of the four Treg clusters at the long-term timepoints (6 months n = 6, 12 months n = 6, 18 months n = 4), demonstrating variability of the clusters among patients. Dots connected by lines indicate individual patients, each color is an individual patient. The small number of patients at each time point limits statistical analysis.

To evaluate if similar unsupervised clustering populations existed beyond the immediate perioperative period, cells from patients with longer-term samples (≥6 months from surgery, n = 10 individual patients with 18 time points) were clustered. Similar to the perioperative clustering of unstimulated cells, this revealed a predominant cluster with an intermediate expression of FoxP3, CD45RA-negative, a high expression of CD25 and a low/intermediate expression of CTLA-4 (longC1_FoxP3^int^) (Figure 6). This clustering, however, identified three small FoxP3^hi^ clusters: longC10_FoxP3^hi^, longC11_FoxP3^hi^ and longC15_FoxP3^hi^. These small populations seemed to cluster by their variable expression of CD45RA, CTLA-4 and RORγt. Unlike within the perioperative period, none of the four clusters demonstrated a significant expression of PD-1 or CD146. Similar to the perioperative samples, the cells in the predominant cluster, longC1_FoxP3^int^, seemed to remain relatively stable across time points, and there was noted variability of the frequencies of the three small FoxP3^hi^ clusters (longC10_FoxP3^hi^, longC11_FoxP3^hi^ and longC15_FoxP3^hi^) among the patients. These data, similar to our findings from standard gating, suggest that the majority of circulating Tregs at the later time points after surgery remain in a predominant, stable cluster with intermediate FoxP3 expression, while small proportions of Tregs with a high expression of FoxP3 are more varied. Unfortunately, the small number of samples at each of the longer-term timepoints prevents meaningful statistical analysis.

### 3.7. Tregs via Unsupervised Clustering of Single-Cell Sequencing from Tumors, Lymph Nodes and Blood

**Figure 7:**
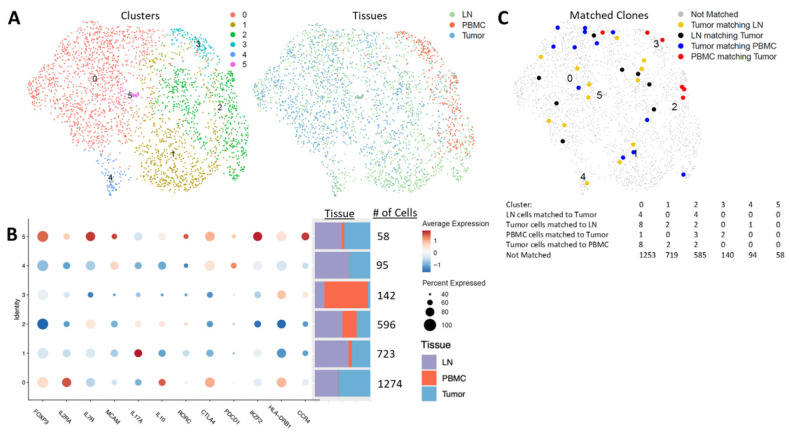
Paired single-cell RNA/TCR sequence clustering reveals six CD4+ Foxp3-expressing clusters within the blood, tumors and lymph nodes. (**A**). UMAPs of unsupervised clustering of patients (n = 5) from single-cell RNA sequencing analysis from combined peripheral blood (PBMCs), tumors and lymph nodes (LNs) of CD4+FoxP3+ cells. The left UMAP demonstrates the six clusters, with each cluster having a corresponding number and color. The right UMAP highlights the tissues that make up each cluster. (**B**). Dot plots showing the relative gene expression of the six CD4+FoxP3+ clusters, the cell number in each cluster and the proportion of cells isolated from lymph nodes (LNs), peripheral blood (PBMC) and tumors comprising each cluster. Circle size corresponds to the percentage of cells in each cluster and the color indicates the relative gene expression (high = red, low = blue). (**C**). UMAP highlighting the numbers of matched clones in each cluster by single-cell TCR sequencing. Colored dots indicate a clonal match corresponding to the associated key, and grey dots indicate the clones that are not matched across tissue types. The table denotes the exact number of matched clones by tissue in each cluster.

Because we did not observe a decrease in the circulating Tregs following surgery, we questioned how similar peripheral blood Tregs were to those found in the TME and regional lymph nodes. To investigate whether circulating Tregs were reflective of the TME, we collected the blood, regional lymph node tissue and tumor tissue of one patient with circulating data and four additional patients (n = 5), and performed scRNA-seq and scTCR-seq enriched with T cells to compare Treg populations across these tissue compartments. Analysis of CD4+FoxP3+ cells revealed six clusters with varying expression of FoxP3 (Figure 7A,B). The largest cluster was predominantly found in the tumor and lymph node, and only minimally in the blood (seqC0_FoxP3^hi^). The cells in seqC0_FoxP3^hi^ were high in the expression of *FOXP3*, *IL2RA* (CD25), *CTLA4*, *HLA-DR* and *IL-10*, and low in *IL7R* (CD127), *PDCD1* (PD-1), *IKZF2* (Helios) and *CCR4*, indicating that these cells are likely activated Tregs. Three clusters with substantial numbers of cells across the blood, tumors and lymph nodes were identified (seqC1_FoxP3^int^, seqC2_FoxP3^low^ and seqC3_FoxP3^int^). seqC1_FoxP3^int^ was the second-largest cluster, present with the highest proportion within lymph nodes, then tumors, and only minimally in the blood. The cells in seqC1_FoxP3^int^ had a relatively low expression of FoxP3, CD25, CTLA-4, HLA-DR, PD-1 and IL-10, and a high expression of IL-17, indicating that these may be pro-inflammatory Tregs induced in an inflammatory microenvironment in these tumor and lymph node tissues [35,36]. seqC2_FoxP3^low^ was predominantly found in lymph nodes, with similar proportions in tumors and the blood. The cells in seqC2_FoxP3^low^ had the lowest expression of FoxP3, CD25, CTLA-4, HLA-DR and Helios, and a moderate expression of IL-17, IL-10 and RORγt (*RORC*), indicating that they may be similar to the Fr III subpopulation identified in standard gating, and may be a mix of Treg and non-Treg cytokine-producing effector cells. 

Interestingly, seqC3_FoxP3^int^ was present at the largest proportion in blood and only in small numbers in tumors and lymph nodes, but with a relatively small total number of cells across the three tissue compartments. The cells in seqC3_FoxP3^int^ had a relatively intermediate expression of Foxp3, CD25 and Helios, low expression of CTLA-4 and PD-1, and a high expression of HLA-DR and CCR4 relative to the other clusters. This suggests that this population may be recently activated and trafficking to the tissue sites of tumor-related inflammation [11,36]. 

The two remaining clusters (seqC4_FoxP3^low^ and seqC5_FoxP3^hi^) were predominantly identified in tumors and lymph nodes. seqC4_FoxP3^low^ was not found in the blood, and the cells exhibited a low expression of Foxp3, CD25, CD127, Helios and HLA-DR, but high expression of CTLA-4, PD-1 and melanocyte cell adhesion molecule (*MCAM* or CD146), indicating that they were likely exhausted. seqC5_FoxP3^hi^ was found at equal proportions in the lymph nodes and tumors, with only a few cells in the blood, and was overall the smallest cluster identified. The cells in seqC5_FoxP3^hi^ had a high expression of FoxP3, CD25, CD127, CTLA-4, HLA-DR, PD-1, CCR4, CD146, RORγt and IL-10, indicating that they are likely recently activated effector Tregs that are trafficking or have trafficked to tissues.

Using scTCR-seq, we evaluated clonal matching across the three tissue compartments (Figure 7C). We found very few clonal matches between the tumor, LN tissues and blood. The highest number of matches was identified in seqC0_FoxP3^hi^. This cluster was composed mostly of cells from tumors and lymph nodes, with only a small number of cells found in the blood. seqC3_FoxP3^int^, which was the predominant blood cluster, had only two clonal matches to cells from the tumor microenvironment. seqC2_FoxP3^low^, which was the most evenly divided across all three tissue compartments, had three PBMC clonal matches to cells from tumors, and two cells from tumors clonally matched to PBMCs. Clusters 4 and 5 had no clonal matches from PBMCs to tumors or lymph nodes. Thus, our scRNA-seq and scTCR-seq data add further evidence to the heterogeneity of Tregs across tissue compartments, and suggest that the serial monitoring of circulating Tregs may be informative, but not completely reflective of the tumor and regional lymph node microenvironments in NSCLC patients.

## 4. Discussion

To evaluate changes in circulating immune cell populations, particularly Treg subsets, in patients undergoing surgical resection for NSCLC, this study used the powerful analytic platform of multi-color flow cytometry, with both standard gating and high-dimensional unsupervised clustering strategies. Our data add to the limited literature related to the changes in circulating immune cells in the perioperative period and beyond, in lung cancer patients. In addition, we used scRNA-seq and scTCR-seq to further characterize Tregs within the tumor, lymph node and peripheral blood compartments.

Our whole-blood leukocyte data demonstrated an immediate and transient increase in the percentage of circulating neutrophils that returned to preoperative levels by 2 weeks following surgical resection. Transient, increased neutrophils corresponded to a significant transient increase in the NLR, but a stable CD4–CD8 ratio in our cohort of patients, indicating the relative stability of total CD4 and CD8 lymphocytes. Increased neutrophil counts and NLRs have been previously reported in the blood of NSCLC patients at the time of surgery compared to age-matched controls [4]. Furthermore, a pre-treatment NLR ≥ 2.5 has been associated with worse survival in NSCLC [5,6]. The clinical significance of an NLR that rises immediately in the post-operative period and returns to preoperative levels requires additional investigation.

Tregs are known to suppress anti-tumor immunity and are thought to have a negative impact on the prognosis of NSCLC. However, the specific role they play in the growth and dissemination of NSCLC remains unclear, as they exist in heterogeneous populations [14,37,38]. Multiple studies have identified circulating Tregs that increase in the blood of NSCLC patients prior to treatment compared to healthy controls [9,10,11,12,13]. In our cohort, flow cytometry standard gating demonstrated the total Tregs and three Treg subsets of NSCLC patients to be within the range reported in the literature [20,32]. We did not find statistically significant changes in total Tregs or the three subsets at 2 weeks, 6 months, 12 months or 18 months following lung resection by standard gating in our cohort of patients. Of note, Kotsakis et al. did not find a significant difference in effector Treg levels between patients with lung cancer and healthy donors [12]. Zhang et al. reported a decrease in circulating Tregs in 98 stage I NSCLC patients at 1, 3, 7, 30 and 90 days following lung resection [39]. However, this study classified CD4+CD25+CD127low cells as Tregs, and did not stain for FoxP3. Similarly, a small decrease was noted by Chen et al. in CD4+CD25+FoxP3+ cells in 36 NSCLC patients 1 to 3 months after surgery [40]. However, it is difficult to determine if the small decrease in these studies is clinically significant, despite being statistically significant. Additionally, it is possible that the relatively small number of patients in our study may have limited our ability to detect a meaningful change in Tregs following surgery. 

Standard gating strategies that fractionate Tregs can be subjective and these Treg subsets are likely made of further subpopulations of cells [32]. Advances in high-dimensional flow cytometry analyses allow for more sophisticated techniques that can replace traditional manual, labor-intensive, operator-dependent, subjective techniques, where cells are gated by evaluating two markers at a time [25,33,34]. In our cohort, unsupervised clustering revealed similar Treg subpopulations to standard gating that clustered by the relative expression of FoxP3 and CD45RA, but at somewhat different proportions. Similarly to a recent study [11], the majority of Tregs identified in our study were CD45RA-negative, consistent with memory/effector Tregs in both standard gating and unsupervised clustering. Unsupervised clustering revealed a significant increase in two small Treg FoxP3^hi^ populations 2 weeks following surgery, that were minimally present prior to resection. Both clusters had a high expression of FoxP3, CD25, CTLA-4 and PD-1. The longer-term data also demonstrated small FoxP3^hi^ Treg clusters, but with a lower relative expression of CD25, CTLA-4 and PD-1. This likely indicates a non-specific response to surgery within the perioperative period, rather than a reflection of a tumor-specific response or a response to tumor excision.

Our scRNA-seq analysis identified six subpopulations of Tregs, with varying expression of FoxP3 across the cells isolated from tumor, lymph node and blood compartments. While the peripheral blood contained most of these cell clusters, several of them were found predominantly, or only, in tumors and adjacent lymph nodes. The largest cluster was identified predominantly in tumors and lymph nodes, with only a small component in the blood, and was consistent with previous reports from NSCLC tumors with a high expression of FoxP3, CD25, CTLA-4, HLA-DR and IL-10, and low expression of Helios [4,38,41,42]. In addition, FoxP3+Helios- Tregs have been reported to be expanded in NSCLC tumors, and are associated with an increased expression of inflammatory cytokines such as IL-17, and with poor survival [43]. The cells from two clusters that were mostly found in tumors and lymph nodes, seqC1_FoxP3^int^ and seqC2_FoxP3^low^, had a lower expression of FoxP3 and Helios, and demonstrated IL-17 expression. The selective deletion of Helios in the TME can contribute to the unstable phenotype of Tregs and their conversion to effector T cells [43]. Thus, the cells in these two clusters may be transitioning to a more effector T cell phenotype, which is further evidence of their plasticity based on their surrounding microenvironment. 

Our scRNA-seq analysis also revealed two clusters not found or minimally found in blood, seqC4_FoxP3^low^ and seqC5_FoxP3^hi^, with high expression of *MCAM* (CD146). MCAM is thought to increase endothelial adherence of T cells to traffic to sites of inflammation and a higher percentage of CD146+ Treg in the blood of lung cancer patients compared to healthy controls has been reported [44,45]. seqC2_FoxP3^low^ and seqC5_FoxP3^hi^ were also noted to have an increased expression of IL7R (CD127). While Tregs are thought to be CD127^low^ relative to other T cells, CD127+ Tregs have been identified and shown to mature into more traditional CD127^low^CD25^high^ Tregs, indicating that CD127 expression is a marker of recent activation in a subset of Tregs [46,47]. As such, our data are consistent with previous reports demonstrating that Tregs exist in heterogenous populations across tissue compartments in patients with NSCLC [14,37,38]. Interestingly, we found very few clonal matches by scTCRseq between tumor and LN tissues and blood. Thus, our scRNA-seq and scTCR-seq suggest that serial monitoring of the peripheral blood for changes in Treg populations can be informative, but may not be completely reflective of the Tregs present in the tumor and local lymph node microenvironments. 

The findings of our study should be interpreted in the setting of several limitations. First, there is a relatively small number of patients and it may not have been powered to detect a statistically significant difference in circulating Tregs with surgery. Second, while all patients were suspected to have early-stage NSCLC, some patients had more advanced disease and two patients did not have NSCLC (one carcinosarcoma and one granuloma). This variability in NSCLC stage and pathology may have created variation that limited our ability to detect a difference with lung resection in our cohort. In addition, patients may have had an undiagnosed pathology, affecting their circulating Tregs that could have influenced our results. Finally, the limited long-term follow-up time points due to the COVID-19 pandemic significantly limit the ability to generate meaningful conclusions from the longer-term data. Nevertheless, we feel that the rigor of our analyses and the addition of unbiased, unsupervised clustering and single-cell sequencing data add significant validity to our findings. 

While we did not find a significant decrease in circulating Tregs after lung resection, this pilot study adds to the limited literature regarding the effects of lung surgery on circulating immune cells, particularly Tregs, and their significant variability among patients with NSCLC. Our scRNA-seq and scTCR-seq data add further evidence to the heterogeneity of Tregs across tissue compartments, and suggest that serial monitoring of circulating Tregs may be informative, but not completely reflective of the local lymph node and tumor microenvironments in NSCLC patients. These findings require further validation with a larger cohort of patients. 

## 5. Conclusions

This pilot study demonstrates significant variability in the circulating levels of immune cells, particularly Tregs and Treg subsets, within the perioperative lung resection period. We noted a transient increase in the percentage of neutrophils and the neutrophil–lymphocyte ratio immediately following surgery, which returned to preoperative levels by 2 weeks. Contrary to our hypothesis, we did not detect a significant change in circulating total Tregs or Treg subsets by standard gating in our small cohort of patients. High-dimensional unsupervised clustering identified similar populations of circulating Tregs, with no significant change in the predominant population following surgery and variability of small FoxP3^hi^ cell populations. Single-cell RNA and TCR sequencing analysis of tumors, lymph nodes and the blood revealed heterogenous Treg populations across tissue compartments, and indicated that Tregs in the peripheral blood only partially reflect populations found in the tumor microenvironment. 

## Figures and Tables

**Table 1:** Clinical characteristics of consenting patients.

Variable	N = 25
Age, Mean (SD)	69.1 (6.6)
Male, (%)	14 (56.0)
Smoking History, (%)	
Never	1 (4.0)
Current	6 (24.0)
Former	18 (72.0)
Pack Years, Mean (SD)	40.2 (28.2)
Pulmonary Function, Mean (SD)	
FEV1 (L)	2.2 (0.5)
FEV1 %	81.0 (13.8)
DLCO %	78.0 (13.1)
Comorbidities, %	
Hypertension	12 (48.0)
COPD	8 (32.0)
Diabetes	2 (8.0)
Oral Steroids	0
Resection Type, (%)	
Wedge	3 (12.0)
Segment	1 (4.0)
Lobe	21 (8.0)
Surgical Approach, (%)	
Minimally Invasive	21 (84.0)
Open	4 (16.0)
Tumor Histology, (%)	
Adenocarcinoma	16 (64.0)
Squamous Cell	5 (20.0)
Carcinoid	2 (8.0)
Carcinosarcoma	1 (4.0)
Granuloma	1 (4.0)
Tumor Size, cm, Mean (SD)	2.6 (1.0)
Pathologic Stage, (%)	
Benign (Granuloma)	1 (4.0)
I	14 (56.0)
II	5 (20.0)
III	5 (20.0)
Lymph Node Status, (%)	
Negative	16 (64.0)
Positive	9 (36.0)

## Data Availability

The data and coding strategies presented in this study are available on request from the corresponding author. The data are not publicly available due to the Dartmouth–Hitchcock policy.

## Supplementary Materials

The following supporting information can be downloaded at: https://www.mdpi.com/article/10.3390/curroncol30050387/s1, Supplementary Table S1: Flow cytometry staining panels; Figure S1: Schematic of patients consenting, enrolled and analyzed; Figure S2: Leukocyte manual gating strategy; Figure S3: Treg subset manual gating strategy; Figure S4: Circulating treg proportions stratified by smoking and COPD.
