# Supplementary material for: The Effect of Lung Resection for NSCLC on Circulating Immune Cells: A Pilot Study
Source: Curr Oncol. 2023 May 17;30(5):5116–34. doi: 10.3390/curroncol30050387 (PMC10217048; doi:10.3390/curroncol30050387)
Supplement: Supplementary file 1 [file curroncol-30-00387-s001.zip › curroncol-2366547-supplementary.pdf]

Supplemental Table S1. Flow Cytometry Staining Panels

Leukocyte panel:

Antibody	Fluor	ZE5 filter	Supplier	Catalog #	Clone	Titer
CD16	BV421	V420	BioLegend	302038	3G8	1:31
CD45	BV510	V525	BioLegend	304036	HI30	1:80
CD4	FITC	B525	BioLegend	317408	OKT4	1:320
CD56	PE	Y577	Miltenyi Biotec	170-081-014	REA196	1:40
CD8	PerCP-Cy5.5	B720	BioLegend	300924	HIT8a	1:320
CD19	PE-Cy7	B750LP	BioLegend	302216	HIB19	1:320
CD14	APC	R670	BioLegend	325608	HCD14	1:20
CD3	APC-Cy7	R775	BioLegend	300318	HIT3a	1:320

Treg panel:

Antibody	Fluor	ZE5 filter	Supplier	Catalog #	Clone	Titer
**Cell surface markers**						
CD45	BUV395	UV387	BD Biosciences	563792	HI30	1:160
CD3	APC-Fire750	R775	BioLegend	344840	SK7	1:160
CD4	BUV661	UV670	BD Biosciences	566003	SK3	1:80
CD45RA	BV711	V720	BioLegend	304138	HI100	1:40
PD-1	PE-Cy7	B750LP	BioLegend	329918	EH12.2H7	1:80
CD25	PE-Dazzle594	Y615	BioLegend	356126	M-A251	1:80
CD146	BV510	V525	BD Biosciences	563255	P1H12	1:20
**Intracellular markers**						
IL-17A	BV421	V420	BD Biosciences	562933	N49-653	1:40
IL-10	PE	Y577	Miltenyi Biotec	130-121-126	JES3-9D7	1:50
CTLA4	BV605	V615	BioLegend	369610	BN13	1:40
FoxP3	FITC	B525	Thermo Fisher	11-4776-42	PCH101	1:40
RORgt	AF647	R670	BD Biosciences	563620	Q21-559	1:40

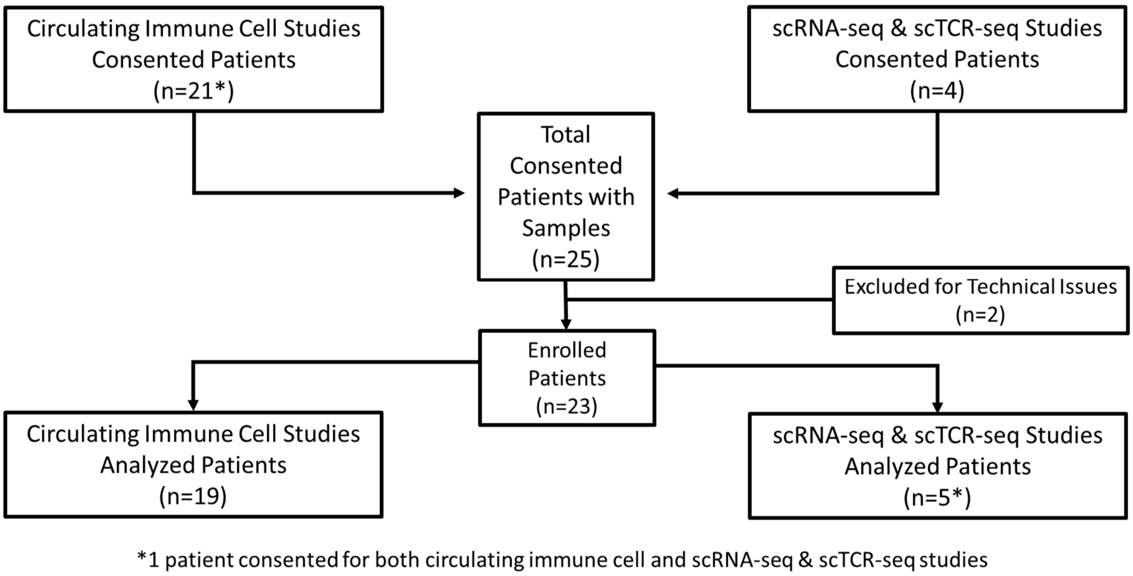

Supplemental Figure S1. Schematic of patients consented, enrolled and analyzed in the study.

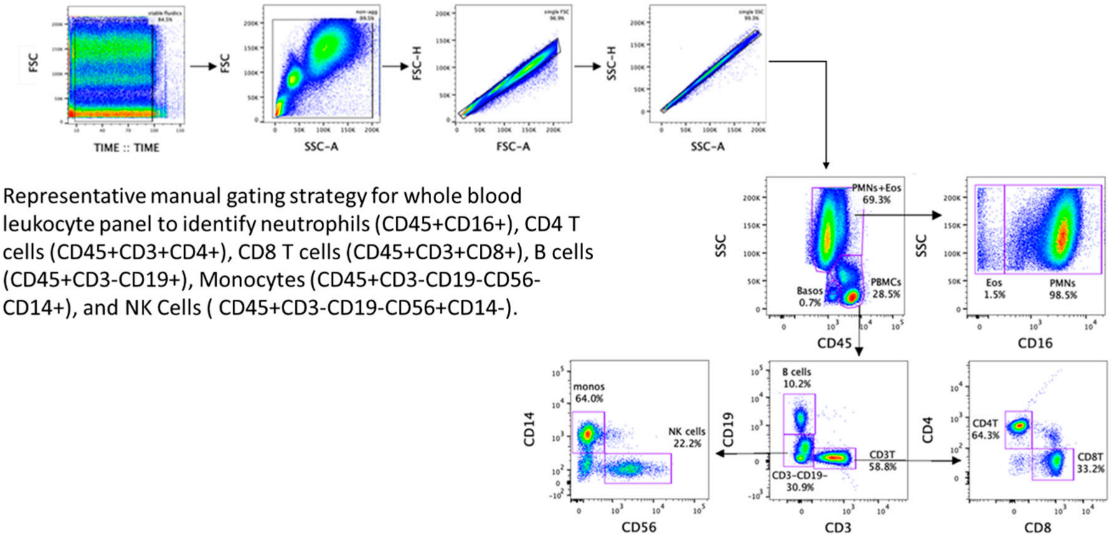

Supplemental Figure S2. Leukocyte manual gating strategy.

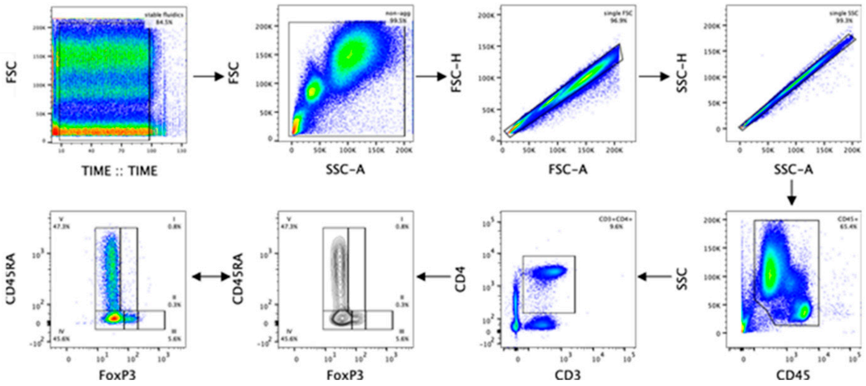

Supplemental Figure S3. Treg subset manual gating strategy.

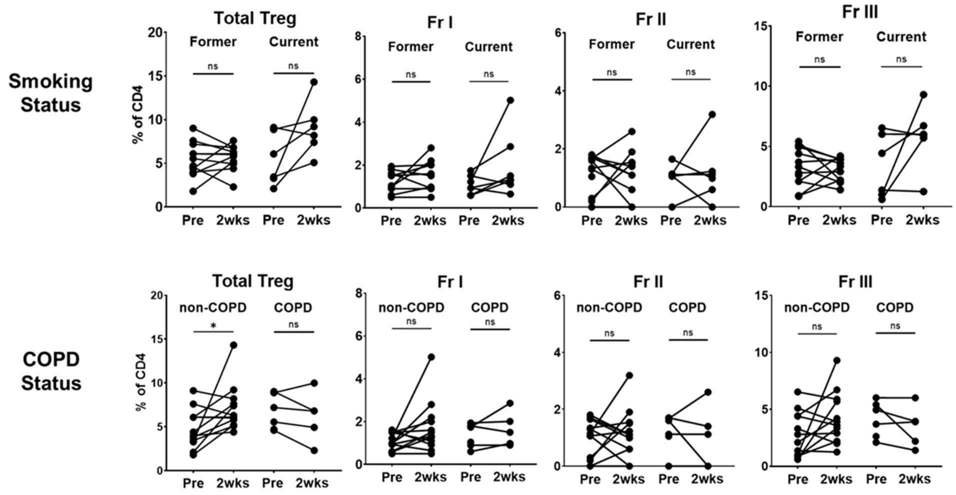

Supplemental Figure S4. Circulating treg proportions stratified by smoking & COPD.
